# Saving the On-Scene Time for Out-of-Hospital Cardiac Arrest Patients: The Registered Nurses' Role and Performance in Emergency Medical Service Teams

**DOI:** 10.1155/2017/5326962

**Published:** 2017-02-09

**Authors:** Ming-Wei Lin, Che-Yu Wu, Chih-Long Pan, Zhong Tian, Jyh-Horng Wen, Jet-Chau Wen

**Affiliations:** ^1^Graduate School of Engineering Science and Technology, National Yunlin University of Science & Technology, 123 Section 3, University Road, Douliou, Yunlin 640, Taiwan; ^2^ChangHua Fire Bureau, 1 Zhongyang Road, Changhua, Changhua 500, Taiwan; ^3^Research Center for Soil & Water Resources and Natural Disaster Prevention (SWAN), National Yunlin University of Science & Technology, 123 Section 3, University Road, Douliou, Yunlin 640, Taiwan; ^4^State Key Laboratory of Hydraulics and Mountain River Engineering, Sichuan University, 24 South Section 1, Yihuan Road, Chengdu 610065, China; ^5^Department of Electrical Engineering, Tunghai University, 1727 Section 4, Taiwan Boulevard, Xitun District, Taichung 407, Taiwan; ^6^Department and Graduate School of Safety and Environment Engineering, National Yunlin University of Science & Technology, 123 Section 3, University Road, Douliou, Yunlin 640, Taiwan

## Abstract

For out-of-hospital cardiac arrest (OHCA) patients, every second is vital for their life. Shortening the prehospital time is a challenge to emergency medical service (EMS) experts. This study focuses on the on-scene time evaluation of the registered nurses (RNs) participating in already existing EMS teams, in order to explore their role and performance in different EMS cases. In total, 1247 cases were separated into trauma and nontrauma cases. The nontrauma cases were subcategorized into OHCA (NT-O), critical (NT-C), and noncritical (NT-NC) cases, whereas the trauma cases were subcategorized into collar-and-spinal board fixation (T-CS), fracture fixation (T-F), and general trauma (T-G) cases. The average on-scene time of RN-attended cases showed a decrease of 21.05% in NT-O, 3.28% in NT-C, 0% in NT-NC, 18.44% in T-CS, 13.56% in T-F, and 3.46% in T-G compared to non-RN-attended. In NT-O and T-CS cases, the RNs' attendance can notably save the on-scene time with a statistical significance (*P* = .016 and .017, resp.). Furthermore, the return of spontaneous circulation within two hours (ROSC_2 h_) rate in the NT-O cases was increased by 12.86%. Based on the findings, the role of RNs in the EMTs could save the golden time in the prehospital medical care in Taiwan.

## 1. Introduction

Emergency medical service (EMS) systems may differ depending on the strategies and the plans in the transportation model and the constitution of the EMS team in different countries [1, 2]. More specifically, for the EMS transportation, the Anglo-American model is based on the “patient to doctor” plan, whereas the Franco-German model is based on the “doctor to patient” plan [3–5]. Different transportation models will create different EMS teams, which may consist of emergency medical technicians (EMTs), nurses, and doctors for the prehospital patient care. In Taiwan, the EMS system follows the Anglo-American model, in which patients are transported to hospitals commonly by two EMTs (referred as 2T) [6, 7].

An EMS case can experience various prehospital time intervals [8], such as the response time [9, 10], the on-scene time [11, 12], and a transportation time. Many researchers emphasize shortening the prehospital EMS time in order to save the golden hours for the patient survival [13–20]. Moreover, the on-scene time will be highly affected by the constituent members of an EMS team, compared to the other time intervals, especially in different EMS models [21, 22].

In some countries, the registered nurses (RNs) had become part of the ambulance service [23]; however, the performance of the RNs in the EMS teams could not be evaluated adequately by a standard metric.

Although different EMS teams have been developed in diverse features for different countries, the performance evaluations of RNs in the EMS teams are still challenging the EMS experts, especially in the critical EMS cases such as out-of-hospital cardiac arrest (OHCA) patients [12].

This research focuses on the evaluation of the effects of the on-scene time by introducing one RN into the original EMS team which is formed by two EMTs. Additionally, it investigates the medical benefits of the saved on-scene time; for instance, the methods of the return of spontaneous circulation within two hours (ROSC_2 h_) and ED length of stay (LOS) are applied to analyze the clinical impact to the OHCA patients.

## 2. Materials and Methods

### 2.1. Study Design

A random participation of the RNs in the EMS teams for prehospital emergency care has been implemented since 2005 at the ChangHua Fire Bureau in Taiwan. In order to evaluate the effects of the RNs in the EMS system, the on-scene time has been analyzed as a primary index to estimate the differentials between these two teams.

The EMS team-attended RNs will receive 46-hour training and participate in programs related to cardiopulmonary resuscitation (CPR), advance cardiac life support (ACLS), and emergency trauma practices. These courses are only offered to nursing personnel with the clinical experience of more than two years.

The RNs accompany two EMTs (referred as 2T1N) in prehospital emergency care from 16:00 to 22:00 in situ every day. When the on-duty RN attends an EMS case, a team of two EMTs (referred as 2T) will be responsible for the subsequent cases until the RN returns to the fire department. On the other hand, once the RN is available, the 2T1N will be teamed again. Accordingly, the participation of the RNs is random, regardless the severity of the EMS cases.

### 2.2. Setting and Selection of Participants

A total of 1375 EMS cases have been collected from the ChangHua Fire Bureau First Battalion Detachment in Taiwan for the period of August 2010 to January 2011; this is a single center study. The EMT keeps the records of dispatch time, scene arrival time, scene departure time, emergency department (ED) arrival time, and ED departure time [8, 9] as well as the patient information, conditions, scene treatments, and so on. In order to eliminate the outliers of the on-scene time of the two EMS teams, 2T and 2T1N, 128 collected cases (9%) which involve residents with difficult accessibility, drunks, and mentally disordered patients were excluded for the sake of undue bias of the on-scene time. The participation rate of the RNs is 55.81% in this study, while the ED time is defined as the time interval between patient arriving and departing from the EDs.

### 2.3. Data Collection and Processing

After the case screening, 1247 patients have been separated into the categories of trauma and nontrauma cases. The nontrauma cases are subcategorized into OHCA (NT-O), critical (NT-C) (OHCA excluded), and noncritical (NT-NC) cases, whereas the trauma cases are subcategorized into collar-and-spinal board fixation (T-CS), fracture fixation (T-F), and general trauma (T-G) cases. This assortment is based on the case records of the official EMS sheets provided by EMTs. The patient percentages of the NT-O, NT-C, NT-NC, T-CS, T-F, and T-G cases are 2%, 12%, 21%, 5%, 5%, and 55%, respectively. The on-scene time is defined as the period from the arrival of the ambulance at the scene until the vehicle leaves the scene [11]. During this time period, the EMS teams implement the prehospital first-aid care to the patients. Additionally, the dispatch time is the point where the EMTs are informed for their cases and depart; the scene arrival time is the point where the ambulance reaches the scene and the EMTs get off; the scene departure time is the point where the EMTs complete the patient transfer to the ambulance and the ambulance departs from the scene; the ED arrival time is the point where the ambulance reaches the ED; the ED departure time is point where the EMTs complete the patient handovers [8, 9]. Each time point can be traced and double-checked by the Global Positioning System (GPS) installed in the ambulances. Nevertheless, there are some slight differences of time records between the EMT run reports and the GPS data.

For OHCA cases, the return of spontaneous circulation within two hours (ROSC_2 h_) rate refers to the percentage of the OHCA patients who have recovered their pulses after the on-scene triage. All the ED data in this research are obtained from the Changhua Christian Medical Center which is responsible for the critical patients in central Taiwan.

### 2.4. Primary Data Analysis

All data are extracted from the records on the official EMS sheets, and the on-scene time is obtained from the ambulance scene-departing time minus scene-arriving time. The mathematic method of the arithmetic mean is applied in the average on-scene time calculation, while the statistical significance is determined by the *t*-test or the *F*-test with *P* < .05.

The ROSC_2 h_ rate of NT-O patients is expressed as the ROSC_2 h_ patients divided by the NT-O patients, including the ROSC_2 h_ patients therein.

## 3. Results

**Figure 1:**
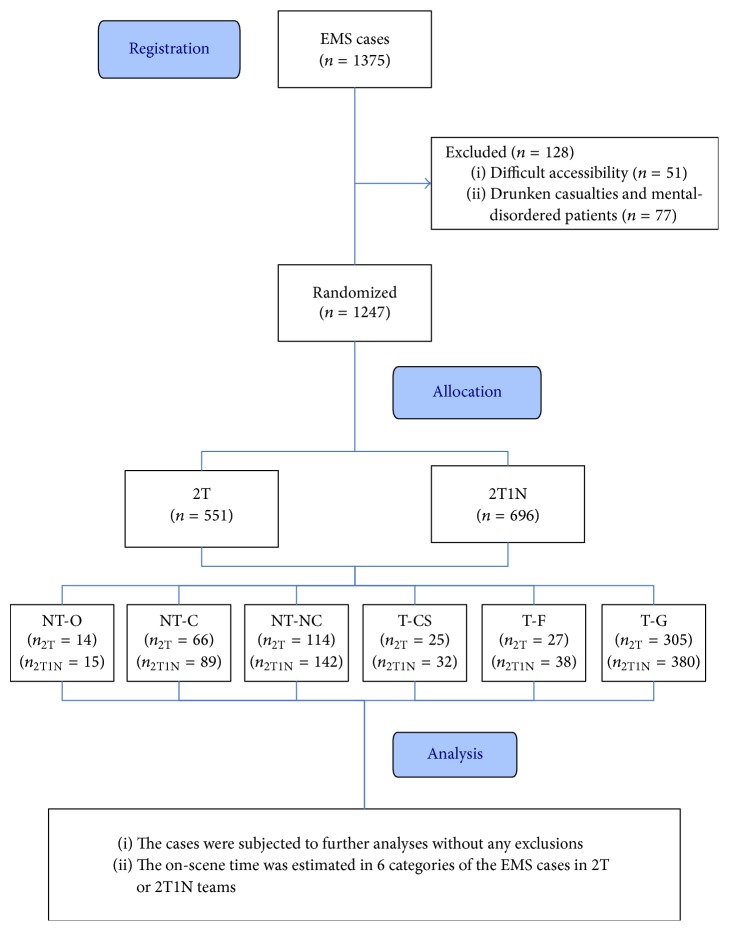
Schematic flow chart of experimental design. 2T, an EMS team with 2 emergency medical technicians; 2T1N, an EMS team with 2 emergency medical technicians and 1 nurse; NT-O, out-of-hospital cardiac arrest (OHCA) patients in nontrauma cases; NT-C, critical patients in nontrauma cases; NT-NC, noncritical patients in nontrauma cases; T-CS, patients with collar-and-spinal board fixation in trauma cases; T-F, patients with fracture fixation in the trauma cases; T-G, general trauma cases.

The patient flow diagram is shown in Figure 1. A total of 51 patients who live in residences with challenging accessibility are excluded from the 1375 EMS cases, because the on-scene time will be overly extended with significant deviations among the cases. For the same reason, drunks and mentally disordered patients, a total number of 77 are also excluded. After the exclusion of the 128 cases, the remaining 1247 EMS cases receive randomly first aid from 2T or 2T1N teams; more specifically, 551 cases were involved in the 2T teams, while 696 cases were involved in 2T1N teams. Moreover, the cases for both teams were subclassified into six categories of NT-O, NT-C, NT-NC, T-CS, T-F, and T-G, and the patient numbers are indicated as *n*_2T_ for 2T and as *n*_2T1N_ for 2T1N teams. Eventually, all the cases of the six categories are subjected to compare the on-scene time between 2T and 2T1N teams.

**Figure 2:**
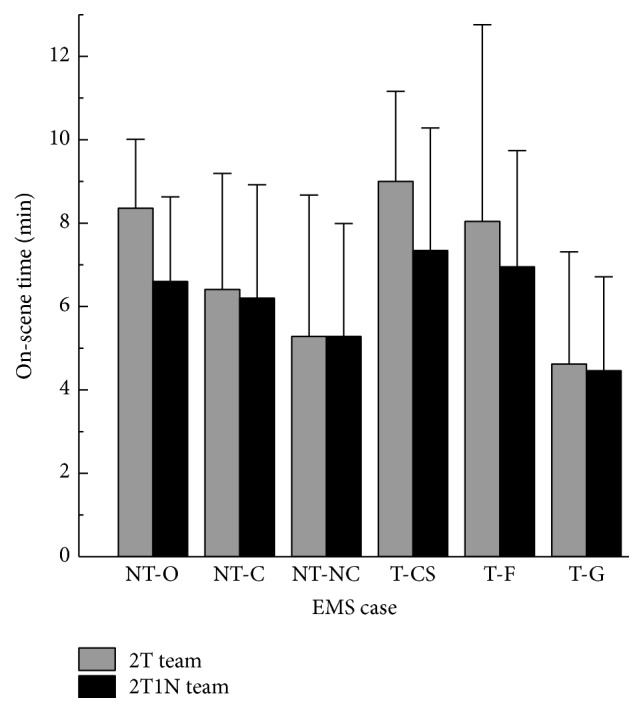
The average on-scene time of the six-categorized EMS cases in 2T and 2T1N teams. 2T, an EMS team with 2 emergency medical technicians; 2T1N, an EMS team with 2 emergency medical technicians and 1 nurse; NT-O, out-of-hospital cardiac arrest (OHCA) patients in nontrauma cases; NT-C, critical patients in nontrauma cases; NT-NC, noncritical patients in nontrauma cases; T-CS, patients with collar-and-spinal board fixation in trauma cases; T-F, patients with fracture fixation in the trauma cases; T-G, general trauma cases.

The average on-scene time of the 2T and 2T1N teams is 6.81 min (with a standard deviation of 2.19 min) and 5.9 min (with a standard deviation of 1.44 min), respectively. For the six types of the EMS cases, the average on-scene time of the NT-O, NT-C, NT-NC, T-CS, T-F, and T-G cases in 2T teams is 8.36 min, 6.41 min, 5.28 min, 9.00 min, 8.04 min, and 4.62 min, whereas in 2T1N teams it is 6.6 min, 6.20 min, 5.28 min, 7.34 min, 6.95 min, and 4.46 min, respectively (Table 1; Figure 2). If the average on-scene time of the 2T teams and the 2T1N teams is compared, a decrease of 1.76 min (21.05%; *P* = .016) in NT-O, 0.21 min (3.28%; *P* = .65) in NT-C, 0 min (0%; *P* = .98) in NT-NC, 1.66 min (18.44%; *P* = .017) in T-CS, 1.09 min (13.56%; *P* = .29) in T-F, and 0.16 min (3.46%; *P* = .39) in T-G cases is noticeable. According to the results of the paired *t*-test for the average on-scene time of the two teams, only NT-O and T-CS cases have a statistical significance (*P* < .05). Regarding the differences of the average on-scene time between these two teams, the 95% of confidence interval in NT-O cases is between 0.36 and 3.16 min, while in T-CS cases it is between −0.26 and 2.44 min.

As shown in Table 2, the ROSC_2 h_ rate of the NT-O cases is 7.14% in 2T teams and 20.00% in 2T1N. Furthermore, the 2T1N teams have an increase of 12.86% of ROSC_2 h_ rate compared to the 2T teams.

Additionally, in order to reveal the interactions between the on-scene time and the ED LOS in 2T and 2T1N teams in six-categorized cases, a statistical method of multivariate analysis of variance (MANOVA) is adopted to perform the *F*-test.

## 4. Limitations

The prime limitation of this research is the uncertain cohort of the 2T and the 2T1N teams. Equally important is the unpredictable situations both teams have to encounter in each EMS case. These limitations will cause internal errors while calculating the on-scene time.

Although the benefits of the attendances of the RNs in EMS can be estimated by using the on-scene time, the medical benefits of 2T1N teams to the patients in total could be evaluated by a comprehensive study, and it could focus on the patient aspects of the medical prognosis, the hospital days, and the survival rate.

Based on the EMS recordings, the cases are classified into six types; however some other types may be more important for understanding the real conditions of the participation of the RNs in the EMS teams.

## 5. Discussion

According to the findings of this research, the 2T1N teams appear to have more medical benefits in saving the on-scene time for EMS system compared to the 2T teams. The average on-scene time is 6.81 ± 2.19 min without attendances of RNs, whereas the average on-scene time is 5.9 ± 1.44 min with the attendances. The average on-scene time has a statistic significant decrease of 21.05% in NT-O and 18.44% in T-CS (*P* = .016 and *P* = .017, resp.). The reason for these discrepancies may be that the RNs can govern the cases, especially when ACLS and intravenous infusions for NT-O patients are needed while the EMTs are performing CPR. In addition to that, the RNs in 2T1N teams can give practical medical advices and decision-making during the on-scene emergency care [23]. Furthermore, the RNs can not only provide emergency medical serves of collar or spinal fixation apparatus to T-CS patients but also facilitate the immobilization of the injured portions for such cases. On the other hand, the NT-C, NT-NC, T-F, and T-G cases require less on-scene treatments, so the RNs in a 2T1N team may detract from effectiveness.

For the NT-O cases, the ROSC_2 h_ rate is 7.14% in 2T teams, while for the 2T1N it is 20.00%. The participation of the RNs reaches a 12.86% promotion in ROSC_2 h_ rate for OHCA patients, which shows that the saved on-scene time and the attendance of RNs could contribute to the real medical benefits of a life-saving purpose [24, 25].

For the sake of the high turnover rate of EMTs in this specific fire department, the study period is limited from August 2010 to January 2011 in order to minimize the effects of the EMT staff discrepancy. During this time period, all the EMTs are the same but may be under different compositions. The nursing staff cannot be controlled in this research, because the cooperative hospital has assigned the RNs programmatically for attending the EMS teams.

Based on the results, all the six-categorized cases show no statistical significance (*P* > .05) between the on-scene time and the ED LOS whether the RNs are attended in the EMS teams or not. The above findings indicate that the ED LOS is an independent event [26] and it is not related to the on-scene time. Therefore, the medical advantages of the attendances of the RNs in the EMS teams should be noticeably evaluated by the methods which exclude the ED time. Nevertheless, the RNs in the EMS teams have a well-communicated practice to report the adequate on-scene physical examinations and triage while transferring the patients to the EDs [23, 27, 28].

Thus far, it remains a challenge to exclude completely the benefits of the RNs attendances in EMS teams from the manpower-added issues. However, the shortage of the on-scene time between the 2T1N and 2T teams is not a solid constant just by reviewing the results of the six-classified cases. The findings of this research may highlight not only the fact that the extra RNs are manpower-added advantages but also medical-based benefits [23, 28]. Based on the EMS practice, the RNs with rich clinical experiences can play a major role in on-scene triage, patient stabilization, leadership assumption, case reporting, decision-making, and first-aid treatment, especially in critical EMS cases [22, 23, 28].

For EMS cases, the on-scene time has a high impact in a compressed window of emergency medical care, while seconds could mean life or death. Therefore, the introduction and participation of RNs in the EMS teams show a plethora of EMS benefits by shortening the on-scene time in the NT-O and T-CS cases and reaching an obvious increase in ROSC_2 h_ rate for OHCA patients.

## Figures and Tables

**Table 1:** The case numbers and the on-scene time of the six-categorized EMS cases in 2T and 2T1N teams.

EMS case	Case number (person)		On-scene time (min)		*P*
	2T	2T1N	2T	2T1N	
NT-O	14	15	8.36 ± 1.65	6.60 ± 2.03	.016
NT-C	66	89	6.41 ± 2.78	6.20 ± 2.72	.650
NT-NC	114	142	5.28 ± 3.38	5.28 ± 2.71	.980
T-CS	25	32	9.00 ± 2.16	7.34 ± 2.94	.017
T-F	27	38	8.04 ± 4.72	6.95 ± 2.79	.290
T-G	305	380	4.62 ± 2.69	4.46 ± 2.25	.390

The EMS cases have been collected from the ChangHua Fire Bureau in Taiwan for the period of August 2010 to January 2011.

The on-scene time is presented as mean ± standard deviation in minute (min), and *P* value is obtained by paired *t*-test.

2T denotes an EMS team containing 2 emergency medical technicians, while 2T1N denotes 2 emergency medical technicians and 1 nurse in an EMS team.

The nontrauma cases were subcategorized into out-of-hospital cardiac arrest (OHCA) (NT-O), critical (NT-C), and noncritical (NT-NC) cases, while the trauma cases were subcategorized into collar and spinal board fixation (T-CS), fracture fixation (T-F), and general trauma (T-G) cases.

**Table 2:** The ROSC_2 h_ rate of the NT-O cases in 2T and 2T1N teams.

NT-O case	Case number (person)	
	2T	2T1N
ROSC_2 h_	1	3
Total	14	15
ROSC_2 h_ rate	7.14%	20.00%

The return of spontaneous circulation within two hours (ROSC_2 h_) rate refers to the percentage of the out-of-hospital cardiac arrest (OHCA) patients who have recovered their pulses after reaching the EDs.

2T, an EMS team with 2 EMTs; 2T1N, an EMS team with 2 EMTs and 1 nurse; NT-O, the OHCA patients in nontrauma cases.
